# Investigating longitudinal changes to frontal cortico-striatal tracts in Huntington’s disease: the IMAGE-HD study

**DOI:** 10.1007/s11682-022-00699-6

**Published:** 2022-06-29

**Authors:** Brendan Tan, Rosita Shishegar, Stuart Oldham, Alex Fornito, Govinda Poudel, Nellie Georgiou-Karistianis

**Affiliations:** 1 School of Psychological Sciences and The Turner Institute for Brain and Mental Health, Faculty of Medicine, Nursing and Health Sciences, Monash University, Clayton Campus, Melbourne, Victoria 3800 Australia; 2 The Australian E-Health Research Centre, CSIRO, Melbourne, Australia; 3 Monash Biomedical Imaging, 770 Blackburn Road, Melbourne, Victoria 3800 Australia; 4 Developmental Imaging, Murdoch Children’s Research Institute, The Royal Children’s Hospital, Melbourne, VIC 3052 Australia; 5 Sydney Imaging, Brain and Mind Centre, the University of Sydney, Sydney, New South Wales 2050 Australia; 6 The Mary MacKillop Institute for Health Research, Australian Catholic University, Melbourne, Victoria 3000 Australia

**Keywords:** Huntington’s disease, White matter, Tractography, Diffusion tensor imaging

## Abstract

The striatum is the principal site of disease pathology in Huntington’s disease and contains neural connections to numerous cortical brain regions. Studies examining abnormalities to neural connections find that white matter integrity is compromised in HD; however, further regional, and longitudinal investigation is required. This paper is the first longitudinal investigation into region-based white-matter integrity changes in Huntington’s Disease. The aim of this study was to better understand how disease progression impacts white matter tracts connecting the striatum to the prefrontal and motor cortical regions in HD. We used existing neuroimaging data from IMAGE-HD, comprised of 25 pre-symptomatic, 27 symptomatic, and 25 healthy controls at three separate time points (baseline, 18-months, 30-months). Fractional anisotropy, axial diffusivity and radial diffusivity were derived as measures of white matter microstructure. The anatomical regions of interest were identified using the Desikan-Killiany brain atlas. A Group by Time repeated measures ANCOVA was conducted for each tract of interest and for each measure. We found significantly lower fractional anisotropy and significantly higher radial diffusivity in the symptomatic group, compared to both the pre-symptomatic group and controls (the latter two groups did not differ from each other), in the rostral middle frontal and superior frontal tracts; as well as significantly higher axial diffusivity in the rostral middle tracts only. We did not find a Group by Time interaction for any of the white matter integrity measures. These findings demonstrate that whilst the microstructure of white matter tracts, extending from the striatum to these regions of interest, are compromised during the symptomatic stages of Huntington’s disease, 36-month follow-up did not show progressive changes in these measures. Additionally, no correlations were found between clinical measures and tractography changes, indicating further investigations into the relationship between tractography changes and clinical symptoms in Huntington’s disease are required.

Huntington’s disease (HD) is an autosomal dominant, neurodegenerative condition that causes catastrophic damage to numerous cellular structures in the brain (De Souza & Leavitt, 2014). Principal amongst these is the striatum (particularly the caudate), which shows pronounced atrophy as the disease progresses (Dominguez et al., 2016; Domínguez et al., 2013; Georgiou-Karistianis et al., 2013b). As such, caudate atrophy is widely considered the most sensitive marker of disease progression in HD (Georgiou-Karistianis et al., 2013b; Tabrizi et al., 2012). However, damage caused by HD is not only limited to the striatum, but also to white matter connections throughout the brain (Domínguez et al., 2013; Dumas et al., 2012; Poudel et al. 2015b; Sweidan et al., 2020). White matter in the brain is comprised of a complex network of axonal fibres that transmit signals between neurons. When white matter structures are disrupted, communication between neurons can become compromised. This disruption has been proposed to relate to the clinical symptoms observed in HD (Poudel et al., 2014). However, the mechanisms underlying this remains largely unknown.

Due to the sheer complexity of white matter connectivity in the human brain, no single technique can completely characterise the neural network (Mori et al., 2005). As such, many techniques have been employed to study axonal changes, including volumetric analyses (Aylward et al., 1998; Jernigan et al., 1991; Paulsen et al., 2010) and more recently, diffusion weighted imaging (Mascalchi et al., 2004; Poudel et al. 2015b; Seppi et al., 2006; Shishegar et al., 2017). Early investigations into white matter changes that occur in HD focused on volumetric loss (Aylward et al., 1998; Jernigan et al., 1991; Paulsen et al., 2006), and demonstrate similar patterns of white matter degeneration as found in other neurodegenerative conditions, such as Alzheimer’s disease (Jernigan et al., 1991) and Parkinson’s disease (Pozorski et al., 2018; Rektor et al., 2018). These studies report that white matter loss is a key feature of HD (Jernigan et al., 1991), with symptomatic HD (symp-HD) individuals showing significantly greater loss compared to those with Alzheimer’s disease. Other studies have found that white matter loss occurs early in the disease process (Tabrizi et al., 2011), beginning in the posterior frontal regions before spreading to other cortices. Additionally, white matter loss has been found to exceed grey matter loss in frontal regions (Aylward et al., 1998), implicating axonal integrity loss as another marker of disease progression.

Diffusion Tensor Imaging (DTI) enables the investigation of microstructural abnormalities in vivo by characterising the diffusion of water in the brain (Jones, 2008), allowing researchers to analyse the integrity of specific neuroanatomical circuits. Inferences about white matter integrity and abnormalities can be made using DTI measures, such as Fractional Anisotropy (FA), Axial Diffusivity (AD) and Radial Diffusivity (RD) (Basser & Pierpaoli, 2011). FA provides detail about the degree to which water diffusion is greater along some directions compared to others, and can be used as a measure of overall axonal integrity (Basser & Pierpaoli, 2011). AD and RD are generally accepted as providing more granular information about white matter microstructure than FA (Song et al., 2003), quantifying the diffusion of water parallel and perpendicular (respectively) to the axonal structure. Whilst some researchers warn of potential problems arising from interpreting AD and RD, as being indicative of underlying changes to myelin and axonal density (Wheeler-Kingshott & Cercignani, 2009), numerous investigations of neuropathological changes have shown the usefulness of using these as in vivo measures of white matter integrity.

In HD, DTI has been used to identify microstructural damage in specific white matter tracts and networks, including striatal motor loops (Georgiou-Karistianis & Egan, 2011), the corpus collosum (Phillips et al., 2013), fronto-caudal connections (Klöppel et al., 2008), and networks involving the prefrontal cortex, motor cortex and putamen (Poudel et al., 2014). Many have reported inconsistent results. For example, some observed significant FA changes in the caudate and putamen (Douaud et al., 2009; Hobbs et al., 2013), whilst others have reported changes in the putamen only (Georgiou-Karistianis & Egan, 2011; Rosas et al., 2006). Furthermore, tractography abnormalities have been associated with motor and cognitive dysfunction (Georgiou-Karistianis & Egan, 2011; Poudel et al., 2014) and the onset of symptoms in HD (Klöppel et al., 2008). These findings suggest that damage to white matter microstructure can be used as a measure to predict functional decline in HD.

Although existing studies have provided a snapshot of neural networks impacted by the disease, no studies have utilised a large longitudinal dataset to examine how these changes occur over time in specific regions within the frontal cortico-striatal tracts (i.e., precentral, rostral-middle frontal and superior frontal regions), in either pre-HD or in symp-HD individuals. Doing so would provide further clarity about the neuropathology of disease, particularly pertaining to how white matter microstructure degenerates over time, and how these changes are related to developing symptoms in HD.

The aim of the current study was to investigate whether white matter tractography changes occur in tracts extending from the caudate to specific cortical regions of interest (ROIs) over-time. Given that previous studies have found the frontal and motor regions of the brain to be primarily impacted by white matter degeneration (Aylward et al., 1998; Klöppel et al., 2008; Poudel et al., 2014; Tabrizi et al., 2011), we chose to investigate specific tracts extending from the caudate to the precentral (the brain region housing the motor cortex), rostral middle frontal and superior frontal regions from the well-validated Desikan-Killiany brain atlas (Desikan et al., 2006). Furthermore, we sought to establish whether tractography changes were associated with worsening clinical symptoms observed in HD.

## Methods and materials

### Participants

A total of 108 participants were originally recruited as part of the IMAGE-HD longitudinal database, comprised of 36 healthy controls, 36 pre-HD and 36 symp-HD (Georgiou-Karistianis et al., 2013a). Controls were matched to pre-HD participants by age and gender. Clinical, cognitive and multimodal neuroimaging measures were acquired at three time-points: baseline, 18-month and 30-month. For this study, 11 controls, 11 pre-HD and 9 symp-HD were excluded from the sample due to incomplete data. Of these 31 participants excluded from the analysis, 28 were lost to follow-up (at 18-months or 30-months) and three were lost to image analysis fails. This left a total of 77 total participants remaining (25 controls, 25 pre-HD, 27 symp-HD). The average age of participants at baseline was 43.89 for controls, 41.13 for pre-HD and 53.20 for symp-HD.

Pre-HD and symp-HD participants underwent gene testing prior to enrolment for the study. CAG repeat length ranged from 39 to 49. A Unified Huntington’s Disease Rating Scale total motor score (UHDRS-TMS) was derived and as per Tabrizi et al., (2009), gene positive participants who scored ≤ 5 were included in the pre-HD group. Those who scored ≥ 5 were included in the symp-HD group. Lifetime exposure to the mutant huntingtin protein was measured by the Disease Burden Score (DBS) expressed as: Age x (CAG—35.5). Demographic and clinical data are included in Table 1 (below).

**Table 1:** Demographic and clinical data for each group, at each time point

		Controls (*n* = 25) Mean ± SD	Pre-HD (*n* = 25) Mean ± SD	Symp-HD (*n* = 27) Mean ± SD
Gender (M:F)	Baseline			
Age (Years)	Baseline	43.88 ± 13.47	41.13 ± 9.71	53.20 ± 9.36
CAG	Baseline	-	42.36 = ± 2.04	42.89 ± 2.26
UHDRS-TMS	Baseline	-	0.92 ± 1.19	18.67 ± 9.81
	18-month	-	2.84 ± 4.04^a^	22.52 ± 11.25^a^
	30-month	-	2.80 ± 4.44	24.07 ± 12.96
DBS	Baseline	-	269.21 ± 59.44	376.84 ± 67.15
	18-month	-	279.69 ± 61.39	388.18 ± 70.23
	30-month	-	287.02 ± 63.01	396.01 ± 72.13
Total Caudate Volume	Baseline	7.59 ± 1.02	6.38 ± 1.29^ cd^	4.84 ± 0.80^c^
	18-month	7.54 ± 1.01	6.19 ± 1.32^ cd^	4.53 ± 0.76^c^
	30-month	7.49 ± 1.01	6.09 ± 1.39^ cd^	4.40 ± 0.79^c^

*SD*, Standard deviation; *UHDRS-TMS*, Unified Huntington’s disease rating scale-total motor score; *DBS*, Disease burden score; ^a^ = Significant from Baseline (*p* = 0.05); ^b^ = Significant from 18-month (*p* = 0.05), ^c^ = Significant from controls (*p* =  < 0.05), ^d^ = Significant from Symp-HD (*p* =  < 0.05)

### MRI data acquisition

MRI scanning was conducted at the Murdoch Children’s Research Institute (Royal Children’s Hospital, Vic, Australia), using a Siemens 3 Tesla scanner. T1-weighted images were acquired for each participant (192 slices, 0.9 mm slice thickness, 0.8 mm × 0.8 mm in-plane resolution, 320 × 320 field of view, TR = 1900 ms, TE = 2.6 ms, flip angle = 9°) (Refer to Domínguez et al., 2013 and Georgiou-Karistianis et al., 2014 for further details).

DTI whole brain images were acquired using double spin echo diffusion weighted EPI sequence (TR = 5800 ms, TE = 82.3 ms, acquisition matrix = 128 × 128, FOV = 24cm^2^, slice thickness 2.5 mm, 50 contiguous axial slices). The diffusion-sensitizing gradient encoding (B1) was applied in 60 directions (b = 1200 s/mm^2^) and 5 images acquired without diffusion weighting (b = 0 s/mm^2^).

### MRI pre-processing

Probabilistic tractography streamlines were generated using MRtrix3. MRtrix3 provides a suite of tools for image processing, analysis and visualisation of white matter using diffusion-weighted MRI (Tournier et al., 2019). Images were pre-processed using the DWI pre-processing pipeline outlined in [https://mrtrix.readthedocs.io/en/latest/dwi_preprocessing/denoising.html]. This included DWI distortion correction, image registration, atlas registration, DWI pre-processing and tissue segmentation.

Atlas registration was completed to provide cortical and subcortical (i.e. caudate) brain regions. This was completed using the well-validated Desikan-Killiany brain atlas, an automated labelling system (Desikan et al., 2006). Note, that this atlas automatically generates maps for each hemisphere separately. As such, all analyses were performed calculating mean values for both hemispheres of each brain region in order to minimise the number of multiple comparisons.

Once pre-processing was complete, images were processed to quantify measures of white matter integrity and generate a visual map of white matter tracts.

### Tractography

Whole-brain tractography streamline reconstruction included brain mask generation, response function generation and streamline generation using “Second-order Integration Over Fiber Orientation Distributions” (iFOD2). “Spherical-deconvolution Informed Filtering of Tractograms” (SIFT) was used to improve the quality of tract reconstruction outlined in https://mrtrix.readthedocs.io/en/latest/quantitative_structural_connectivity/sift.html. White matter tractography measures were generated for the tracts of interest using subcortical and cortical parcellations from the Desikan-Killiany brain atlas as noted in the section above.

### White matter tractography measures

The mean fractional anisotropy (FA), axial diffusivity (AD) and radial diffusivity (RD) were calculated as the mean value of all fixels (fibre bundles within each voxel), within each of the four regions of interest. The directionality of diffusivity (as measured by AD and RD) can provide more granular information into the nature of axonal damage or demyelination occurring in HD.

#### Fractional Anisotropy (FA)

FA is a metric that provides a simple and robust measure of the degree of anisotropic diffusion of water occurring within a region (Smith et al., 2012). Because FA reflects the degree of anisotropic diffusion, it will be high (i.e., approaching unity) in regions of high organization (e.g., corpus callosum), intermediate in regions with some degree of organization (e.g., white matter regions that have no strong predominant axon fiber axis orientation), and low in tissues where the predominant cell shape, and therefore diffusion, is not specifically oriented (e.g., grey matter) and approaching zero in free fluids (e.g., CSF) (Pfefferbaum et al., 2000). Reductions in FA are associated with axonal damage.

#### Axial Diffusivity (AD)

AD is a metric which quantifies the diffusion of water parallel to white matter fibres. Whilst a decrease in AD is indicative of axonal damage (Song et al., 2003), an increase in AD may represent axonal degeneration and loss due to neuronal trimming (Beaulieu, 2002).

*Radial Diffusivity (RD)* RD is a metric which quantifies the diffusion of water perpendicular to white matter fibres. An increase in RD is indicative of axonal damage (Song et al., 2005) and reflective of demyelination (Beaulieu, 2002).

### Statistical analysis

Using IBM SPSS Version 24.0 for Windows, comparisons of tractography measures (FA, AD, RD), were conducted for within groups (baseline, 18-month, 30-month) and between groups (controls, pre-HD, symp-HD) using a general linear model (repeated measures ANCOVA) with Group and Time as Independent Variables (IVs) and each tractography measure (i.e., FA, AD, RD) as the dependent variables (DV). Age and gender were used as covariates for all analyses.

Summary statistics were calculated for the three groups on each of the white matter integrity measures and for each cortical region at each time point. Significant differences between groups and time points were calculated using t-tests. Bonferroni corrections were used to adjust for multiple comparisons across the ROIs and tractography measures.

### Correlation analysis

Pearson’s correlations were performed between clinical measures (i.e., UHDRS-TMS and DBS) and tractography measures for each region of interest. A correlation analysis between baseline tractography measures and UHDRS-TMS was conducted in the symp-HD group only. Correlations between change in mean cortical tractography measures and DBS was conducted for both HD groups.

### Post-hoc analyses

Post-hoc partial correlation analyses were conducted to investigate the correlation between caudate volume change (TP 3 minus TP1) and change in white matter tractography changes (FA, AD, RD), controlling for age and gender.

## Results

Using a general linear model (repeated measures ANCOVA), we sought to determine whether white matter integrity changes (FA, AD, RD) occurred in specific tracts from the caudate to regions of interest (Precentral Gyrus, Superior Frontal and Rostral Middle Region), over time in pre-HD and symp-HD, compared to controls.

### Main effects and Interaction effects on FA

There were no significant interaction effects of Group by Time in any of the regions of interest.

**Fig. 1:**
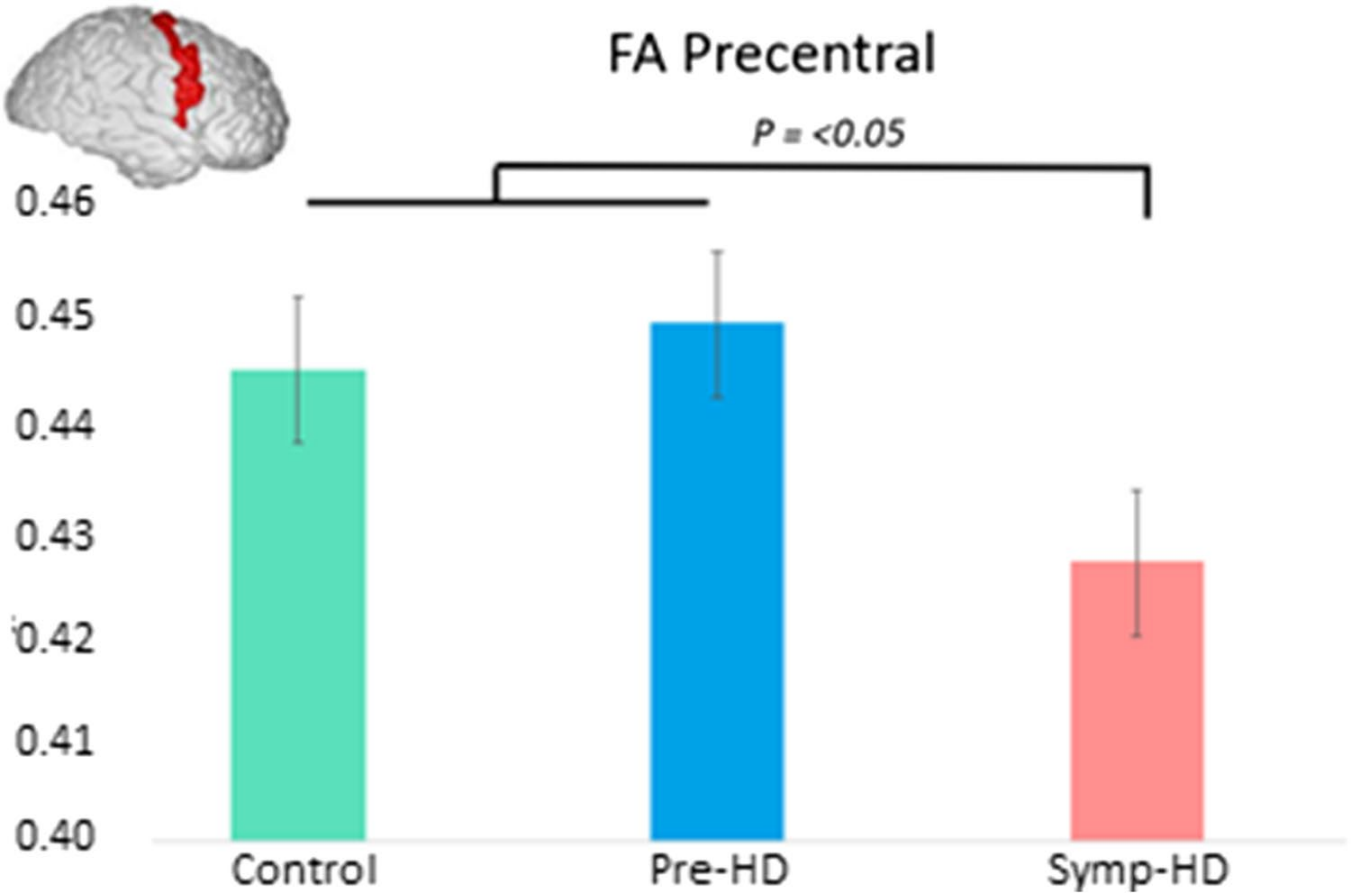
Mean FA in the precentral region across groups

Significant main effects of Group were found in the Precentral region, *F* (2,72) = 3.20, *p* = 0.047. However, after correcting for multiple comparisons, this did not remain significant. Controls and pre-HD showed significantly higher FA than symp-HD in this area (see Fig. 1). There were no significant effects of Group in the Rostral Middle or Superior Frontal regions.

There were no significant main effects of Time in any regions of the interest.

### Main effects and Interaction effects on AD

There were no significant interaction effects of Group by Time on AD in any of the regions of interest.

**Fig. 2:**
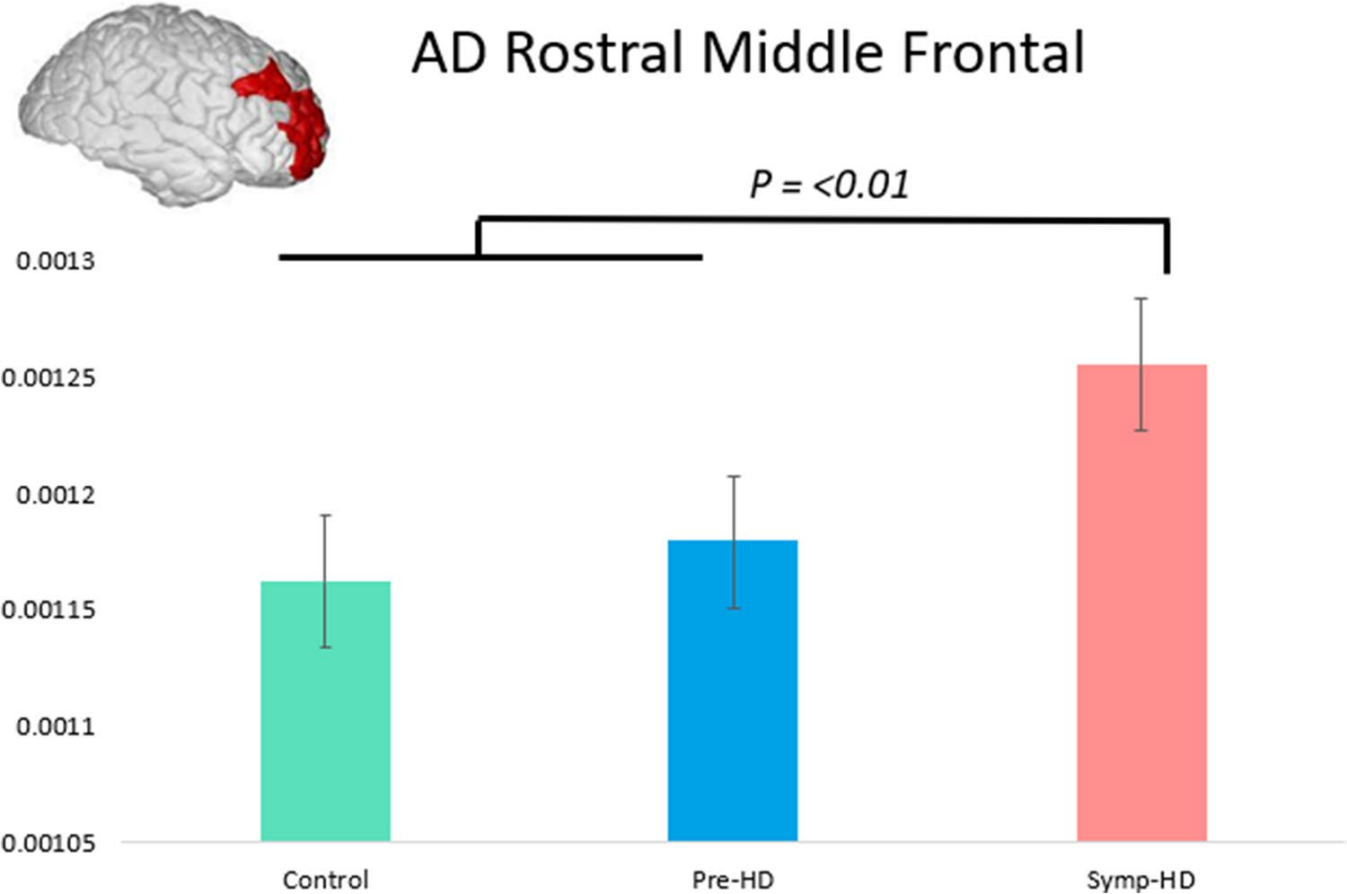
Mean AD in the rostral middle frontal region across groups

Significant main effects of Group were found in the Rostral Middle Frontal region, *F* (2, 72) = 9.83, *p* =  < 0.01. Controls and pre-HD showed significantly lower AD than symp-HD individuals (see Fig. 2).

There were no significant main effects of Time on AD in any of the regions of interest.

### Main effects and interaction effects on RD

There were no significant interaction Group by Time effects in any of the regions of interest.

**Fig. 3:**
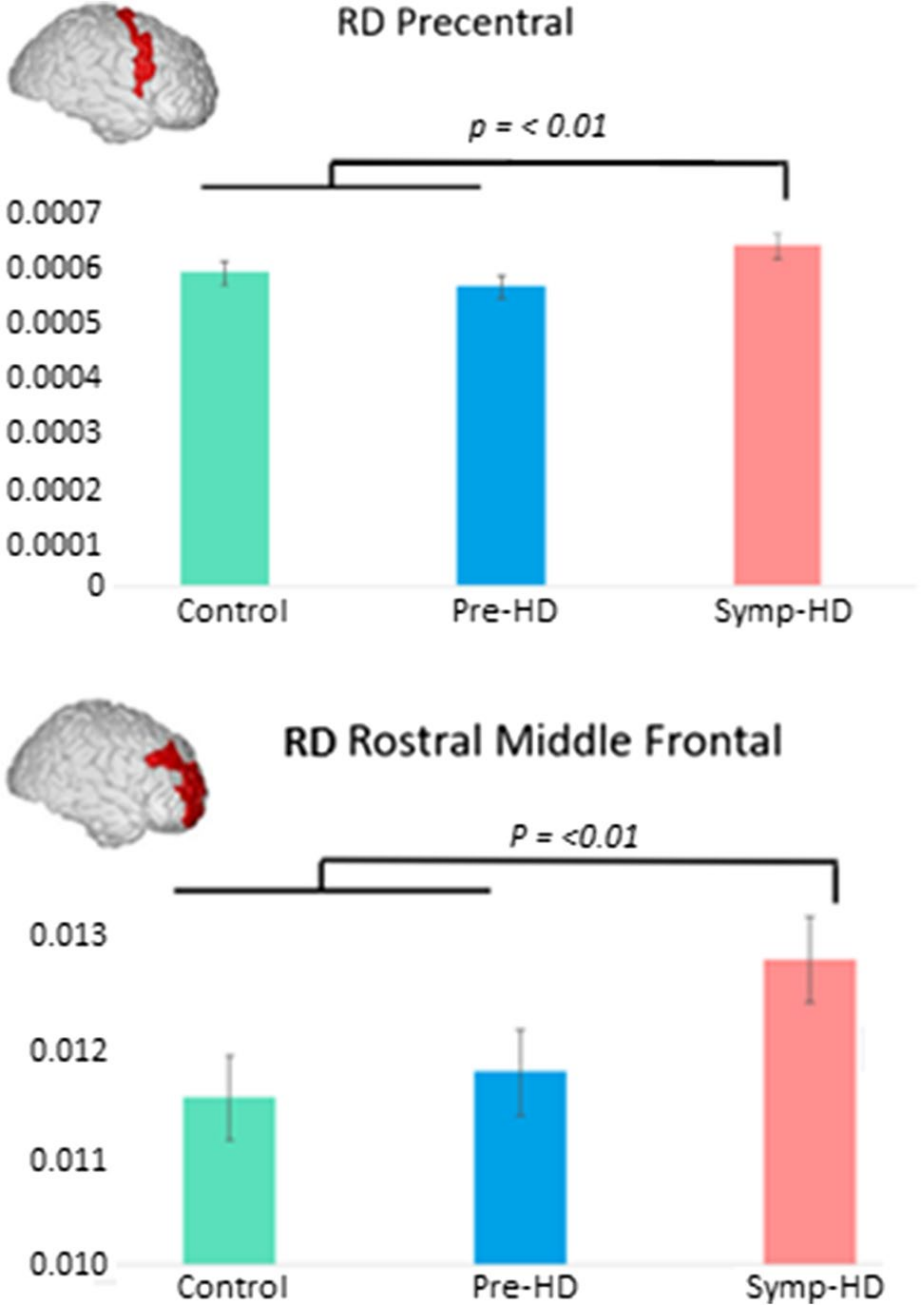
Mean RD in the precentral and rostral middle frontal regions across groups

There were significant main effects of Group in the Precentral, *F*(2,72) = 3.39, *p* =  < 0.05 and Rostral Middle Frontal Regions, *F*(2,72) = , *p* =  < 0.01. In the Rostral Middle Frontal region, symp-HD individuals had significantly higher RD than controls and re-HD. In the Superior Frontal region, symp-HD differed significantly from controls, but not from pre-HD. See Fig. 3.

There were no significant main effects of Time in any of the regions of interest.

### Correlations between clinical and tractography measures

#### Correlations between change in UHDRS-TMS and white matter integrity measures

To investigate the relationship between white matter integrity and motor symptoms, partial correlations between change in UHDRS-TMS (score at 30-months minus score at baseline) and change in white matter integrity measures were conducted in the symp-HD group only. There were no significant correlations between change in UHDRS-TMS and change in white matter integrity measures in any of the eight regions of interest.

#### Correlations between baseline DBS and change in white matter integrity measures

Partial correlations between baseline DBS and change in white matter integrity measures (FA, AD, RD, score at 30-months minus score at baseline) were calculated with both pre-HD and symp-HD groups combined to determine the impact of exposure to the disease across the disease continuum. There were no significant correlations between baseline DBS and change in white matter integrity measures in any of the eight regions of interest.

#### Correlations between CAG repeat length and change in white matter integrity measures

Partial correlations between CAG repeat length and change in white matter integrity measures (FA, AD, RD, score at 30-months minus score at baseline) were calculated with both pre-HD and symp-HD groups combined to investigate the relationship between CAG repeat length on tractography changes. There were no significant correlations between CAG repeat length and change in white matter integrity measures in any of the eight regions of interest.

#### Correlations between change in caudate volume and change in white matter integrity measures

Partial correlations between change in caudate volume and change in white matter integrity measures (FA, AD, RD, score at 30-months minus score at baseline) were calculated in all groups to determine the relationship between caudate volumetric changes and white matter integrity. After controlling for age and gender, a significant negative correlation between change in caudate volume and RD in the rostral middle frontal region only (*r* (76) = -0.325, *p* =  < 0.01).

## Discussion

The primary aim of this study was to examine longitudinal white matter tractography changes in the caudate and precentral, superior frontal and rostral middle frontal brain regions in pre-HD and symp-HD, compared to controls. We did not find a significant interaction effect of Group by Time for any measure in the three regions of interest, nor were there any significant effects of Time. However, we demonstrated significant group differences of FA in the precentral frontal region, AD in the rostral middle frontal region, and RD in the precentral and rostral middle frontal regions with the symp-HD group showing significant differences compared to the pre-HD and control groups (the latter two groups did not differ from one another). Tractography changes were not correlated with change in UHDRS-TMS or DBS in any of the regions of interest.

The finding of significant group level tractography changes in symp-HD from the caudate to the precentral region supports previously established findings that motor circuits are particularly vulnerable in HD (Georgiou-Karistianis & Egan, 2011; Poudel et al., 2014). We found that the symp-HD group had significantly decreased FA, compared to pre-HD and controls, indicating that tracts to the precentral region are likely affected during the symptomatic stage of disease. As HD is primarily a motor disorder, and clinical diagnosis is determined by the development of motor symptoms, it would be expected that white matter connections to the motor cortex would be compromised in symp-HD. Indeed, others have reported similar compromise of the structural connectivity of motor circuits (Georgiou-Karistianis & Egan, 2011; Poudel et al., 2014). In this study, we measured an approximate ~ 4.5% reduction in FA from the caudate to the motor cortex in symp-HD, compared to the FA at baseline, to ~ 2.5% reduction at 30-months. It is unclear why the difference in FA between controls and symp-HD reduced over this time period, but this could be indicative of either selective neurodegeneration or adaptive neuroplasticity (De Erausquin & Alba-Ferrara, 2013; Mole et al., 2016).

Elevated AD was observed in the tracts radiating from the caudate to the rostral middle frontal region in conjunction with significantly increased RD to the precentral and rostral middle regions in symp-HD. These findings were somewhat expected as previous studies had found increased AD and RD in the corpus callosum (Phillips et al., 2013; Rosas et al., 2010) and whole brain (Weaver et al., 2009) in symp-HD, compared to controls (Liu et al., 2016). Whilst an increase in AD can indicate an increased coherence of tracts, this may also be suggestive of a selective neurodegenerative process occurring in symp-HD, which results in fewer neural branches. Alternatively, higher RD may indicate diffusion of water molecules perpendicular to the direction of the tract, potentially reflecting impaired myelin. The rostral middle frontal region houses the dorsolateral prefrontal cortex, which is integral for executive functions including working memory, which is well documented as being impaired in symp-HD compared to controls (Georgiou-Karistianis et al., 2014; Poudel et al. 2015a). Although we did not investigate the relationship between working memory performance and tractography measures in this paper, future studies could utilise this as a measure of functional decline. Together, these findings characterise the potential damage to white matter tracts caused by HD; namely selective neurodegeneration in the rostral middle frontal region accompanied by demyelination of white matter tracts in the precentral and rostral middle frontal regions.

Volumetric change in the caudate is a well-established neuropathological feature in HD (Georgiou-Karistianis & Egan, 2011; Domínguez et al., 2013). We reported a significant negative correlation between change in caudate volume and change in RD in the rostral middle frontal region only, such that as total caudate volume decreases, radial diffusivity to the rostral middle frontal region increases. It is possible that the findings of the present study are a secondary consequence of the degenerative process influencing caudate volumetric changes, rather than a demonstration of novel degenerative patterns in white matter tractography per se. For example, atrophy in the caudate caused by the *m*HTT protein may also influence a secondary pruning effect of white matter tracts projecting from it. Alternatively, decreased volume in the caudate may also occur in part due to there being fewer tightly packed axonal tracts preventing the radial diffusion of water molecules. However, previous studies have reported the effect of *m*HTT on cerebellar-striatal circuitry prior to the onset of disease (Tereshchenko et al., 2020; van der Plas et al., 2019), indicating that the level of connectivity to, and volume of the striatum are closely linked. However, as we only observed this correlation on one measure, in tracts to the rostral middle frontal region only, it is possible that our findings represent novel degenerative patterns, rather than simply reflecting caudate volume change.There were no significant associations between changes in tractography measures and change in UHDRS-TMS, DBS or CAG repeat length in pre-HD or symp-HD in any cortical regions, despite mean UHDRS-TMS being significantly different between time points. Change in tracts to cortical regions not investigated may be responsible for influencing the clinical presentation of symptoms measured by the UHDRS-TMS. One such region may be the superior parietal lobe, which is responsible for visuomotor control and motor planning (Andersen et al., 1987; De Renzi 1982). Damage to the superior parietal lobe has been found to result in motor deficits (Fogassi & Luppino, 2005) and it is possible that white matter connectivity changes to this region may contribute to the motor signs in HD.

There were certain limitations of the present study. Although we observed some significant group differences, we did not observe a significant effect of Time or any interaction effects of Group by Time. Additionally, numerous neuroimaging studies have reported degeneration in cortical and sub-cortical structures many years prior to clinical diagnosis, and prior to the presence of overt motor symptoms in HD (Dumas et al., 2012; Klöppel et al., 2008). As such, it was surprising that we were unable to detect significant differences between pre-HD and controls. One explanation for this finding is that whilst group level differences occur during the symptomatic stages of the disease, it is likely that these changes occur over a longer period than 30-months. To better understand the nature of tractography changes in these regions, longer intervals between time points are required, as the length of time from baseline to 18-months and 18-months to 30-months may be inadequate to detect such differences. Another possible explanation is that our measures were not sensitive to subtle white matter changes that occur along these tracts over and above the ageing process, especially in the pre-symptomatic group. However, these measures have been shown to successfully detect changes in white matter microstructure in 12 to 18-month longitudinal studies of Alzheimer’s disease (Kitamura et al., 2013; Mayo et al., 2017). Alternatively, the sample size may have been insufficient at detecting small but significant changes occurring in white matter tracts. Thus, larger samples may be required to detect small effects.

## Conclusion

Our 30-month longitudinal study remains the only longitudinal analysis of white matter microstructure changes in both pre- and symp-HD. Although we were unable to identify any interaction effects of Group by Time, which was the primary aim of the study, we did find white matter tractography differences in symp-HD in both the precentral and rostral middle frontal regions, brain regions responsible for motor control and working memory, respectively. Specifically, the symp-HD group had decreased FA in the precentral region, increased AD in the precentral region, and increased RD in the precentral and rostral middle regions compared with the pre-HD and control groups. Although these findings are suggestive of demyelination and selective neurodegeneration that occurs in specific frontal-striatal tracts during the symptomatic stages of disease, longitudinal changes were not detected in the 30-month period observed in this study..

## Data Availability

All data and materials were collected as part of the IMAGE-HD study and are available upon request.
